# Assessment of Genetic Diversity and Conservation in South African Indigenous Goat Ecotypes: A Review

**DOI:** 10.3390/ani12233353

**Published:** 2022-11-29

**Authors:** Aletta Matshidiso Magoro, Bohani Mtileni, Khanyisile Hadebe, Avhashoni Zwane

**Affiliations:** 1 Department of Animal Sciences, Tshwane University of Technology, Pretoria 0001, South Africa; 2 Agricultural Research Council, Animal Production, Irene 0062, South Africa; 3 Agricultural Research Council, Biotechnology Platform, Onderstepoort 0110, South Africa

**Keywords:** adaptation, conservation, genetic variation

## Abstract

**Simple Summary:**

This review seeks to understand the scientific research on the genetic diversity of the indigenous goat ecotypes of South Africa and to provide an understanding of the relationships among the ecotypes and their structure. It is essential for their utilization and for the establishment of conservation priorities. The conservation of genetically diverse ecotypes is significant for sustainable production and relevant breeding objectives. It is, therefore, important to review the knowledge that has been gathered on the genetic diversity and conservation of indigenous goat ecotypes to be able to identify the gaps that remain and the niches for future research. The review focuses on the status of indigenous goat ecotypes in South Africa, the genetic diversity that exists, and the production potential and adaptation by highlighting the importance of prioritization for conservation and sustainable utilization.

**Abstract:**

Goats were amongst the first livestock to be domesticated more than 10,000 years ago for their meat, milk, skin, and fiber. They were introduced to Southern Africa by migrating nations from Central Africa to the south. Due to local adaptation to the different agro-ecological zones and selection, indigenous goats are identified as ecotypes within the indigenous veld goat breed. Their ability to thrive in a resource-limited production system and in challenging environmental conditions makes them valuable animal resources for small-scale and emerging farmers. They play important roles in household agriculture and cultural activities as well as in poverty alleviation. Studies have described the phenotypic and genetic variations in indigenous goats, targeting the major goat-producing regions and the breeds of South Africa. In turn, information is restricted to certain breeds and regions, and the experimental design is often not adequate to inform the conservation status and priorities in changing environments. Advances in genomics technologies have availed more opportunities for the assessment of the biodiversity, demographic histories, and detection regions associated with local adaptation. These technologies are essential for breeding and conservation strategies for sustainable production for food security. This review focuses on the status of indigenous goats in South Africa and the application of genomics technologies for characterization, with emphasis on prioritization for conservation and sustainable utilization.

## 1. Introduction

The African goat population accounts for at least 41% of the world’s goat population, which equates to approximately 423 million goats, of which, 35 million come from Southern Africa [1,2]. South Africa is the third-highest goat-producing country in Southern Africa, preceded by Tanzania and Malawi. These numbers comprise both commercial and indigenous goat breeds. South Africa alone has approximately 5.2 million goats, with a provincial distribution ranging from 0.5% in Gauteng to 38% in the largest producing province, which is the Eastern Cape [2]. The majority of indigenous goats are owned and kept by smallholder farmers in rural areas [3] and are mostly kept for the value they bring to the livelihood of rural communities, through products such as meat, milk, skin, and mohair [4], and through their role in religious and cultural ceremonies.

**Figure 1:**
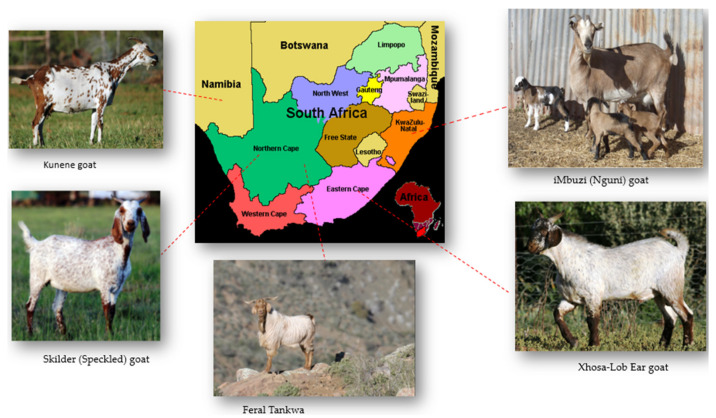
Established Indigenous veld goat ecotypes of Southern Africa and places of origin.

Indigenous goats have diversified into unique ecotype populations over time due to geographical isolation, environmental dynamics, and selection pressures [4]. As a result, they are named after their place of origin, physical characteristics, and/or the ethnic tribes that own them [5]. For example, the iMbuzi (Nguni) originates from Kwa-Zulu Natal, the Xhosa-Lob Ear originates from the Eastern Cape, the Skilder (Speckled) originates from the Northern Cape, the Kunene originates from the Kunene region in Namibia (Kaokoland) [5,6], and the Feral Tankwa goat that is currently located in Tankwa Karoo National Park, in the Northern Cape, South Africa, (Figure 1) [7]. Under circumstances where the ecotypes are uncharacterized and undescribed, they are often referred to as village ecotypes according to the originating community [8]. Together with their crosses, these ecotypes have evolved to adapt to predominantly agro-ecological conditions [9]. These adaptive traits are important for climate change and make goats an important animal resource that needs to be prioritized for conservation for future food security in changing environments [10].

South African communal farming is disadvantaged by herds with fewer animals and a low flock density per population [11]. In most instances, the mating is uncontrolled, undefined, and random, with undefined breeding objectives, resulting in genetic dilution. These factors also put indigenous animals at risk of inbreeding depression, which leads to decreased fitness in a population [11]. Smallholder farmers have less access to improved breeding [12] and therefore rely on bucks through communal grazing, or farmers may borrow each other’s bucks for a certain period of time [11]. This method also leads to crossbreeding, inbreeding, and genetic dilution, and, in a worst-case scenario, it can lead to the extension of a particular breed or ecotype [12]. Thus, it is significant to learn about the importance of conserving the genetic diversity of local indigenous goats to preserve them for sustainable utilization in changing environments.

Genetic characterization is important for the conservation of livestock animals [12]. Characterization is the description of traits that are typically inherited and describes the phenotype and genotype of an animal [13]. Analyses of genetic diversity provide an understanding of the relationships that exist among breeds as well as the within-breed differentiation that constitutes their sustainable utilization and prioritization for conservation [14]. The use of genetic and genomic technologies can assist in identifying populations for conservation through genetic diversity studies and can assist in identifying traits of economic importance [15]. They enable the identification of specific genes and variants and determine their functions in the genome as well as the associations between phenotype and genotype in response to environmental and selection pressures. This information can be used in breeding and in-breed improvement for sustainable production [16]. This review will discuss the status of the characterization and conservation of South African indigenous goat ecotypes as well as technological advances, with emphasis on prioritization for conservation and sustainable utilization.

## 2. Production Potential of South Africa’s Indigenous Goat Ecotypes

Indigenous veld goats (IVG) are found in all parts of Southern Africa, with at least four established ecotypes [17]. They are known for their excellent ability to walk long distances for forage and water and to graze and browse on a wide variety of plants, shrubs, and grasses [5]; their high fertility and long breeding seasons; their excellent herding ability; and their ability to protect themselves from predators with their horns [18]. IVG ecotypes include the Mbuzi (Nguni-type), Xhosa-Lob Ear, and Speckled (Skilder), which originate from South Africa, and the Kunene ecotypes originating from Namibia. The description of Southern Africa’s indigenous goat ecotypes can be found in [18]. The history of the Xhosa-Lob Ear has shown that this ecotype, along with the robust dapple-colored male goat, was used to develop the Boer Goat by breeders in the Eastern Cape, which resulted in the Buffelsfontein Boer Goat Stud [18]. A study by [19] indicated that the Xhosa-Lob Ear was also used in the development of the commercial Kalahari Red. Similar to other indigenous goats, these goats are resistant to most diseases and have the ability to survive drought with no supplementary feeding [20]

The Speckled ecotype, also known as the Skilder, has a high resistance to heat and sunlight. A study by [18] highlighted that Skilders are equivalent to or superior to some South African goat breeds in terms of production. In the study of [17], it was illustrated that IVGs (Speckled and Xhosa-Lob Ear) have a similar potential for meat production under similar production conditions as the Boer Goat. The IVG bucks appeared to be particularly suited for meat production, having a higher meat yield that is leaner and with lower subcutaneous and intramuscular fat than Boer Goats. 

A study on the growth performance of indigenous goats under village systems and improved management systems was conducted [21]. The study showed that when farmers practice control over reproduction, health, nutrition, and husbandry, goat production improves. Good management and record keeping are mandatory when it comes to the improvement of indigenous goats (Table 1). A recent study by [22] established that, although the indigenous village ecotypes have lower growth performance, their growth performance is relatively comparable when they are raised in an intensive production system. The study further showed that the village goats that were raised in intensive production systems performed better than those raised in extensive production systems. This shows that the production of the indigenous village goats can be improved with an improved production system (Table 2). Most farmers suggested that crossbreeding with commercial goats improved the growth performance of the indigenous ecotypes. However, [22] indicated that management alone is able to improve the performance of indigenous goats.

## 3. The Availability of the Illumina Goat SNP BeadChip

Small ruminant genetic advancement has been accelerated as a result of improved management and the use of estimated breeding values. The introduction of single nucleotide polymorphism chips opened the door to genomic selection in many countries for small ruminants [23]. The advancement of genetic technologies has sparked interest in genome-wide studies, mostly those regarding the adaptive and reproductive qualities in small ruminants from developing countries [23]. In 2011, the International Goat Genome Consortium (IGGC) developed the first SNP chip for domestic goats, the Goat SNP50K BeadChip, which contained approximately 53,347 SNPs. The IGGC ‘s principal goal was to develop a medium- to high-density genotyping tool for current domestic goats [24]. The SNPs were developed from goat breeds such as the Saanen, Alpine, Creole, Boer, Kacang, and Savanna. However, studies such as [9,25,26,27] validated the use of this BeadChip on indigenous goats and on other goat breeds that were excluded during the development of the Goat SNP 50K BeadChip. It is important to note that prior to the consolidation, three independent SNP discovery programs were carried out, and variants of goat species were found using different pipelines, one for dairy and mixed breeds, and the other for meat breeds [24,25]. Although other breeds were not included in the development of the BeadChip, high levels of polymorphism have been observed in the excluded breeds, constituting the significant use of the SNP50K BeadChip on other goat breeds [24,25]. The latest vision of the SNP BeadChip is the Goat_IGGC_65K_v2, which is version 2 of the GoatSNP50k Illumina chip containing 59,727 SNPs (http://www.goatgenome.org/projects.html, accessed on 8 October 2022). 

## 4. Genetic Diversity Studies in Southern African Indigenous Goats

Research on goats has taken a turn with the development of new molecular genetic technologies. Microsatellite markers were used for the first time in earlier studies on South African goats, primarily involving genetic diversity and characterization studies [17]. Using microsatellites, the study by [28] established the genetic variation between three commercial (Boer goat, Kalahari Red, and Savanna goat) and three non-descriptive indigenous goat populations from the provinces of Eastern Cape, Mpumalanga, and Limpopo. They showed a high level of heterozygosity in indigenous goats compared to the commercial breeds and indicated the lowest genetic variation in the Boer goat. However, the microsatellite markers had limitations when it came to genetic characterization and the determination of the phylogenetic relationship due to their low variance in the genome [29]. 

The goat Single-Nucleotide Polymorphism (SNP) array later became commercially available and has become the marker of choice for genomic and genomic studies on goats [24]. SNPs are now the preferred markers and represent a reliable method for studying the genome-wide genetic diversity in livestock because they are more informative [24,30]. These markers have enabled studies to determine allele frequencies in populations, allowing for estimations of the level of genetic diversity [31]. The availability of the Illumina Goat 50K SNP BeadChip has presented an opportunity to differentiate between breeds, to perform genome-wide association studies, and to identify markers and genes of economic importance [9,24,25,32]. This is needed for breed improvement to increase production as the demand for food increases. Whereas genetic diversity has a direct impact on genetic-trait-selection methods and for the management of inbreeding, genetic characterization aids in the protection of the distinctive characteristics of indigenous populations [16]. 

Genetic characterization is carried out to determine the degree of polymorphisms present in populations using allelic frequencies that are normally influenced by phenotypic characteristics [14]. However, both genetic and phenotypic characteristics are modified by environmental conditions [33]. In genetic diversity studies, the probability of two alleles chosen randomly from a population is a common factor that is used to quantify variation [34]. Genetic diversity can also be measured by estimating the degree of allele fixation, as measured by the fixation index (*Fst*), which shows the genetic distinction between populations [35]. This is one of the most popular statistical methods used to define population structure and diversity [16]. As of present, few studies (Table 3) characterizing Southern Africa indigenous goat ecotypes using SNPs to determine their genetic variation, relatedness, signatures of selection, landscape genomics, and pathway analysis to understand the genetic adaptation of indigenous goat populations have been carried out [9,32,33]. In the study by [9], SNP markers were used to study the South African indigenous village ecotypes Nguni, Venda, Xhosa, Zulu, and Tswana from the provinces of Eastern Cape, KwaZulu-Natal, Limpopo, and North West. The study identified high levels of genetic diversity and very low levels of inbreeding and linkage disequilibrium in the village ecotypes. Table 1 shows some of the studies that have been conducted in Southern African indigenous goats using microsatellites and SNP markers.

The genetic diversity studies on Southern African indigenous goat ecotypes have shown the usefulness of SNP markers, especially regarding the distinctiveness of the goat breeds/ecotypes, which might have resulted from natural or artificial selection. Natural selection promotes the survival and fitness of individuals within a species. In coming generations, this may result in conservation through purifying selection or through the emergence of new beneficial traits [39]. However, despite their use in genetic diversity studies, SNP markers also enable the analysis of the introgression, demographic, and ancestral history of populations [9,40,41].

## 5. Adaptation of Indigenous Goat to Local Environments 

Goats were among the first animals to be domesticated and have displayed extraordinary adaptability and usefulness [42]. During domestication, an animal undergoes a dramatic shift in physiological and behavioral stress as it transitions from a high-density and disease-prone environment to one created by humans [43]. Candidate genes for selection during goat domestication have been found through studies [44]; however, they change over time with the domestication process, evolution, and adaptation [45]. These transitions have made these animals strive and adapt to different agro-ecological regions, and they have developed traits that enable them to survive and produce. Farming systems in South Africa are characterized by hot, dry, and cold conditions with limited feed, lack of water supply, and high disease prevalence [46]. However, these indigenous breeds and goat ecotypes have naturally adapted to these harsh environments [12,47]. Studies have indicated that goats respond better to environmental heat stress than other livestock animals [48,49]. They can cope better, even when farmed with other livestock species [1]. Due to various morphological, phenotypic, and largely genetic traits, they can adapt to any environmental condition [50]. 

Physical characteristics such as coat color and texture, long legs, and lobby ears provide goats with unique abilities to survive in different climatic conditions and influence their performance in various stocks [51]. Their physical attributes also allow them to be tolerant to diseases, have excellent thermoregulation capacity, and to survive on low-quality forage and during water shortages [12,52]. They have the ability to absorb water from their feces in the rectum and to concentrate their urine as a way to retain water back in their body for survival [53]. Their horns are an important adaptive element for self-defense against predators and to assist them when they have to compete for food and water with other animals and during mating [33]. These adaptive features, including reproductive and productive traits, are linked to natural and artificial selection. Directional natural and artificial selection events have left footprints across the genome known as signatures of selection, which influence adaptation [44]. These signatures can now be unraveled to understand the mechanisms of adaptation and production in different environments. Due to the ever-changing conditions caused by climate change, it is important to characterize and conserve these animals. 

## 6. Conservation Strategies for Goats

For many years, conservation biologists have used population genetics to apprehend threats to endangered species [54], with the most popular markers used being microsatellites. Array-based techniques using SNPs are the most used technology that can genotype numbers of animals with thousands of SNPs, and the genotype information can be used to differentiate between populations, demographic, and structure analysis, and to identify selection signatures [24,30,55,56]. Whole-genome sequencing technologies are also available to discover new SNPs and to unravel the functions of the variants in the genome [57]. By incorporating genomic technologies, conservation genetics is broadening the scope of its studies by overcoming many conservation-related limitations [58]. This could result in a more accurate estimation of genetic variation within and between populations, which will improve our understanding of how the environment and genes interact to affect the phenotype and fitness of species [58]

Many conservation-related concerns that have previously been difficult to solve can now be resolved thanks to the simple ability of genetic markers to improve the efficiency and precision of estimating numerous crucial conservation parameters [16]. The Food and Agriculture Organization (FAO) established that over a third of domesticated farm animals are either extinct or facing the threat of extinction [59] and that these farm animal genetic resources (FAnGR) need to be conserved, especially considering the increase in population growth in the Southern African region [60]. Currently, the Southern African region is facing the challenge of preserving distinctive farm animals that have the potential to contribute to future livestock development while simultaneously trying to upsurge the productivity of the livestock sector [61]. Therefore, it is important to focus the conservation of FAnGR on smallholder farmers because over 90% of animal keepers in Southern Africa are classified as smallholders. Additionally, approximately 75% of farm animals are kept by rural communities [62]. These animals have to meet the primary needs of the rural communities, where they represent a source of food and finance, whereas the second primary need is socio-economic and social events [61].

The first step to setting conservation priorities for the FAnGR is to identify breeds that contribute greatly to global genetic diversity [63]. The breeds must be able to meet future demand and development as well as cultural, social, and religious roles [64,65]. The priority for FAnGR is to conserve breeds that are under threat of extinction [66]. Setting priorities for FAnGR through genetic diversity enables the breed’s ability to adapt to different environmental challenges, meet the market’s supply and demand, have increased production through selection, and respond to disease resistance [67]. Studies that have characterized indigenous goats at the production level [68], genetic level [4,9,32], and phenotypic level [32,33] have been conducted. These studies go a long way in terms of assisting in identifying breeds that constitute conservation. The focus should currently be directed towards the conservation of pure indigenous goats, as the current breeding programs and structures are not considered to conserve genetic diversity. Most breeders are more interested in exotic breeds and crossbreds, which put indigenous breeds in great danger [56]. In response to the FAOs’ Global Plan of Action for the conservation of animal genetic resources, the Department of Agriculture, Land Reform, and Rural Development (DALRRD) established a National Plan for conservation and the sustainable use of FAnGR in 2015 [56] for the conservation of indigenous animals. The National Plan’s objectives were to promote the conservation of genetic resource diversity in animals and to implement cost-effective breeding programs in order to improve animal genetic resources for sustainable animal production systems as well as to contribute to the conservation and use of agricultural ecosystems and the utilization of animal genetic resources [65]. Regardless of the presence of the Global Plan of Action and national plans for the management and utilization of genetic resources, there is still an urgent need for the conservation of indigenous goats, especially in rural areas where there is minimal or no controlled breeding [56]. Recently, it has become even more apparent to conserve indigenous goats, particularly in these current times of unpredictable climate change and global pandemics. The global pandemic that recently occurred could illustrate conditions that justify conservation in particular. During this time, most livelihoods were affected by the lack of food supply, and food security was immediately threatened (https://www.mzansiagritalk.com/archives/7312, accessed on 5 July 2022). Additionally, the heavy rainfalls that took place in parts of South Africa recently resulted in the loss of many animals and potential disease outbreaks to communal and rural farmers. For these reasons, conservation methods, strategies, and plans need to change to conserve animals that will respond to current and future environmental challenges. 

A potential approach as a conservation method developed for smallholder farms is community-based livestock breeding programs (CBBPs). CBBPs are implemented to improve smallholder farmers’ indigenous knowledge as well as to ensure training, competence, and institutional intervention [69]. They provide a sustainable option for the conservation of local animal genetic resources (AnGRs) in their natural habitat by utilizing and improving them [69]. A study in Malawi and Uganda by [15] was conducted to evaluate the practicality of CBBPs as a possible method for conservation and for the development of indigenous small ruminants using case studies of goat CBBPs. Through the preservation of their existing communal pasturelands and the development of pasture production skills, the program encouraged smallholder farmers by giving them access to small ruminant feed resources [15]. The implementation of CBBPs contributed by enhancing the overall animal performance, which resulted in higher offtake rates and pricing by the smallholder farmers [15]. The goat CBBPs in Malawi and Uganda indicated potential in sustaining rural livelihoods and in enhancing the diversity of local goat genetic resources. Goat CBBPs were recently introduced in South Africa by organizations such as the Agricultural Research Council with funding support from the South African government. They had an impeccable influence on the improvement and conservation of indigenous livestock (https://www.mzansiagritalk.com/archives/7312, accessed on 5 July 2022). CBBPs are a good alternative in smallholder production systems with low flock density and little to no financial advantage. 

Different methods have been applied in livestock conservation, mainly to increase production and performance, e.g., crossbreeding, artificial insemination, and so forth. However, setting priorities is the most effective way to implement a significant conservation approach given that the breeds are ranked according to their conservation importance. A study by [70] used Weitzman’s approach to determine the genetic contribution and extinction probability of African cattle breeds. The study outlined the marginal diversity and contribution of each breed to the total diversity. They were able to determine the risk of extinction in African cattle and their environmental context. To the breeds that showed a risk of extinction, positive impacts were observed when the conversation was introduced. Furthermore, populations with a smaller population size had an increased extinction probability. However, increased extinction did not affect their diversity contributions. This shows that breeds that are at threat of extinction are able to contribute greatly to the total diversity, and when conservation methods are applied, the extinction probability is reduced [70]. 

In the study of [71], the extinction probability was calculated based on the contribution of each breed by looking at the area, total breed size, population trend, economic importance, and distinctiveness. Due to different levels of breed significance, the conservation program was applied to breeds of high value and with excellent characteristics such as high productivity and good-quality meat and milk. However, the study by [72,73] suggested that the degree of extinction alone could constitute conservation due to the unpredictable future due to climate change. It was also emphasized that species that currently make low contributions to the economy might be of good use in the future, and conservation measures must be in place to save genetic resources. 

There are currently two types of conservation methods, in situ and ex situ. The ex situ method involves the preservation of genetic material, with animals being removed from their habitats, and it includes cryogenic preservation. The in situ conservation method, also referred to as on-farm conservation, is when animals are conserved within their natural production system. In this method, breeds are developed according to their characteristics [62]. This method accommodates entire agro-ecosystems, as the most useful species are saved as they occur. The breeds that are conserved under in situ methods are able to advance and adapt to changing environmental conditions, making it possible for researchers to establish their unique genetic makeup [74]. However, developments in genomic technologies have now enabled the genetic characterization of these breeds to be studied for conservation and to identify genes of economic importance, including those found in selection signatures regions. 

## 7. The Need to Conserve Indigenous Goat of South Africa 

Goat production plays an important role in the livelihoods of small farming communities, and the adaptive traits of indigenous goats are important for climate change and affordable maintenance [56]. The studies that have been conducted on the genetic diversity, population structure, and adaptation of the indigenous goats of South Africa [3,4,9,27,33,75] have indicated healthy and high levels of genetic diversity in studied populations. Although some [4,9,28] have indicated a substandard population structure amongst village/ non-descriptive goats, their gene diversity and unique genetic background show healthy genetic diversity and adaptation traits. This constitutes them for conservation. Their genetic resources should be conserved to maintain ecological balance for future generations, as the world’s population is increasing, and so is the consumption of natural resources. The need to conserve resources is growing to ensure future sustainable utilization, biodiversity preservation, and ecological balance [76]. The conservation of indigenous goats could significantly contribute to high goat production, which is beneficial to the South African economy [77]. 

## 8. Natural Selection of Indigenous Goats 

The adaptive and phenotypic responses of goats to their production environments and different climatic conditions have led to variation in allelic and genotypic frequencies, contributing to the breed’s diversity through selection [55]. Selection imprints selection signatures in the genome and results in high genetic differentiation within and between populations [78]. Selection signatures are the main driving force of genetic variation and play an important role in understanding the mechanisms of natural selection.

Signatures of selection could be defined as the decline, rejection, or change of genetic variation in genomic regions. It is adjacent to causal variants and responds to natural or artificial selective pressures [30,72,73]. Humans first influenced selection during domestication and the development of new breeds [79]. Insights into the biological pathways underlying phenotypic variation can be determined through the identification of genomic regions subjected to selection. Selection signatures are important in the evolutionary history of breeds and can be used in the identification of genes of economic importance that can be used to improve the breeds [79]. Different methods are available to identify selection signatures in populations. The most popular approaches are thorough estimations of Fst, runs of homozygosity (ROH), XP-EHH (cross-population extended haplotype homozygosity), and haplotype-based within-population iHS (integrated haplotype score) [80,81]. A study by [82] identified regions of selection signatures that affect variation in coat color, growth, and milk composition in Swiss goats using the CaprineSNP50k BeadChip. Other studies detected selection signatures related to adaptation to hot/dry environments as well as regions linked to production and reproduction [80,83].

A study by [84] investigated the genetic diversity of the South African indigenous goat population and identified five genes. *UHRF2*, *GLDC*, *NDST3*, *CFAP61,* and *CUBN* genes were discovered (Table 4). It was established that the *UHRF2* gene was involved in the regulation of biological processes, including metabolic processes, growth processes, and reproduction, whereas the *GLDC, NDST3*, *CFAP61,* and *CUBN* genes were found to be involved in the pathways that are responsible for disease response, thermoregulation, metabolism, and longevity. This highlights the unique adaptability traits of indigenous goats and their significance when it comes to environmental adaptation through selection. A study on the multiple genomic signatures of selection in goats and sheep indigenous to a hot arid environment was conducted by [80]. Several genes that indirectly influenced traits for adaptation to hot arid environments were identified. The genes identified enabled adaptations such as thermo-tolerance (melanogenesis), body size and development, energy and digestive metabolism, and nervous and autoimmune responses. These kinds of analyses make it possible to comprehend the worldwide livestock domestication process and the adaptations of breeds, including goats and goat ecotypes. Genes that play a role in the differentiation of breeds can also be identified [80].

## 9. Conclusions

Population growth, economic development, and the need for food security underline the justification for conserving the animal genetic resources of indigenous farm animals. Indigenous goats are an important part of cultural heritage and contribute to the livelihood of rural communities and need to be protected and conserved. The use of the indigenous goats to improve the commercial breeds and vice versa has neutralized the genetics of indigenous goats. Hence, it is important to genetically characterize these animals using available genomics technologies to conserve and identify priority populations and to preserve them from threats of extinction. These animals may struggle to survive in harsh South African environments, and, therefore, breeding strategies must be changed to conserve animals that will withstand changing climatic conditions. More CBBPs are needed as potential conservation methods. The CBBPs have been shown to have potential for conservation in Southern African countries such as South Africa, Malawi, and Uganda, thus indicating that this strategy can be a success in SADC regions. It has the potential to be implemented and to conserve all of the ecotypes that exist in the SADC. The participation of farmers in these programs will permanently establish institutions and organizations that support goat CBBPs, providing opportunities and guarantees for the sustainable utilization of indigenous goats. 

## Figures and Tables

**Table 1:** Performance of indigenous goats raised in the village and improved management systems [21].

Reference	Indigenous Goats	Village Management (kg)	Improved Management (kg)
[21]	Birth weight (kg)	N/A	1.7
	Weight at: 3 months	6.8	9.2
	Weight at: 6 months	10.0	12.4
	Weight at: 12 months	13.0	20.0
	Weight at: 18 months	17.3	24.1
	Weight at: 24 months	21.5	29.5

**Table 2:** Three different growth stages of village goats under two different production systems [22].

Reference	Breed	Production System	Trial	12 W (kg)	24 W (kg)	36 W (kg)
[22]			1	9	22.5	32
	Village ecotypes	Intensive	2	10.5	19.5	31
			3	16.4	24	33
		Mean ± SD	-	11.97 ± 3.91	22.00 ± 2.29	32 ± 1.00
			1	7	16	22.5
	Village ecotypes	Extensive	2	13	20.5	28
			3	13	15	27.5
		Mean ± SD	-	11.00 ± 3.46	17.17 ± 2.93	26 ± 3.04

**Table 3:** Genetic diversity of Southern African indigenous goats using microsatellite and SNP markers.

Country	Populations	Markers	Marker Density	H_o_	H_E_	Reference
South Africa	Indigenous goats	Microsatellite	10 Microsatellite loci	0.64–0.6981	0.631–0.673	[28]
	Communal indigenous goat populations	SNP Markers	The Illumina Goats 50K SNP BeadChip	0.39–0.42	0.38–0.40	[4]
	Nguni, Tswana, Venda, Xhosa, Zulu, and Tankwa	SNP Markers	The Illumina Goat 50K SNP BeadChip	0.35–0.41	0.33–0.40	[9]
Namibia	Ovambo Caprivi Kunene Kavango	Microsatellite	18 Microsatellite loci	-	0.623	[36]
Mozambique	Pafuri, Tete, Maputo	Microsatellite	17 microsatellites loci	0.553	0.620	[37]
Botswana	Tswana goats	Microsatellite	12 microsatellite loci	0.121	0.162	[38]
	Tswana goats	SNP Markers	The Illumina Goat 50K SNP BeadChip	0.419	0.423	[32]

H_O_, observed heterozygosity; H_E_, expected heterozygosity.

**Table 4:** Estimated chromosomes and genes under selection using F_ST_ [84].

Genes	Fst	Chromosome	Gene Function
UHRF2	0.72	8	Ubiquitin-like with PHD and ring finger domains 2
GLDC	0.69	8	Glycine decarboxylase
NDST3	0.68	6	N-deacetylase and N-sulfotransferase 3
CFAP61	0.68	13	Cilia and flagella associated protein 61
CUBN	0.67	13	Cubilin

## Data Availability

There were no new data analysed for this study.
